# Survival of *Campylobacter jejuni* Co-Cultured with *Salmonella* spp. in Aerobic Conditions

**DOI:** 10.3390/pathogens11070812

**Published:** 2022-07-20

**Authors:** Nagham Anis, Laetitia Bonifait, Ségolène Quesne, Louise Baugé, Wissam Yassine, Muriel Guyard-Nicodème, Marianne Chemaly

**Affiliations:** 1 Unit for Hygiene and Quality of Poultry and Pork Products, Laboratory of Ploufragan-Plouzané-Niort, ANSES, 22440 Ploufragan, France; nagham.anis@anses.fr (N.A.); laetitia.bonifait@anses.fr (L.B.); segolene.quesne@anses.fr (S.Q.); louise.bauge@anses.fr (L.B.); marianne.chemaly@anses.fr (M.C.); 2 Faculty of Sciences, Lebanese University, Beirut 10999, Lebanon; wissamyassineul@outlook.com

**Keywords:** *Campylobacter jejuni*, *Salmonella*, co-culture, survival, poultry production

## Abstract

*Campylobacter* and *Salmonella* are responsible for the two major foodborne zoonotic diseases in Europe; poultry is the main infection source. *Campylobacter* cannot grow under aerobic conditions, but can show aerobic survival when co-cultured with other microorganisms; however, its interaction with *Salmonella* has not been studied yet. In this study, these two bacteria were co-cultured under controlled aerobic conditions. Different concentrations and strains of *C. jejuni* were incubated with or without different *Salmonella* serotypes (10 CFU) at 37 °C for 16 h. *C. jejuni* did not grow after incubation with or without *Salmonella*. The survival of *C. jejuni* was observed only for the highest initial concentration of 6 log CFU/mL with or without *Salmonella*. However, its survival was significantly higher when co-cultured with *Salmonella*. No survival was observed at lower concentrations. *C. jejuni* survival was positively affected by the presence of *Salmonella* but depended on the *Salmonella* serotype, the *C. jejuni* strain and the initial concentration. On the other hand, the *Salmonella* enumerations were not affected by *C. jejuni*. Our results suggest potential interactions between *Salmonella* and *C. jejuni* that require further investigations for a clearer understanding of their behavior in natural habitats.

## 1. Introduction

*Campylobacter* and *Salmonella* are both responsible for the two major foodborne zoonotic diseases in Europe with 120,946 and 52,702 cases, respectively, reported in humans in 2020 [1]. A reduction in the number of cases was reported in 2020 compared with 2019, when more than 220,682 and 87,923 cases of campylobacteriosis and salmonellosis, respectively, were reported [2]. This decline was probably due to the COVID-19 pandemic and the withdrawal of the United Kingdom from the EU [1]. However, the overall EU trend for confirmed human cases of these two diseases in 2016–2020 showed no statistically significant increase or decrease [1]. Campylobacteriosis and salmonellosis are the two most commonly reported causes of bacterial gastroenteritis in many high-income countries, affecting millions of people every year [3,4]. In addition, they lead to a major public health and economic burden worldwide [2,5]. There are common signs and symptoms of both diseases, including fever, diarrhea, abdominal pain, nausea and sometimes vomiting [6,7]. In few cases, infection with *Campylobacter* can evolve into severe complications such as the Guillain-Barré syndrome, which is an acute autoimmune neuropathy with ascending paralysis [8]. According to a study conducted in France between 2008 and 2013, the annual number of hospitalizations was estimated at 5182 for campylobacteriosis and 4305 for salmonellosis [9]. Poultry is generally recognized as the main source of infection with *Campylobacter* [10,11] and *Salmonella* [12]. Moreover, these two pathogens are frequent inhabitants of the digestive tract of poultry—whose intestines offer a suitable biological niche for their survival and dissemination—although these bacteria do not affect poultry health [13,14]. Transmission to humans occurs primarily through the ingestion of contaminated poultry products, undercooked chicken meat or products, and cross-contaminations during the handling of meat in the kitchen [7,15]. Moreover, raw eggs and food prepared with raw eggs are also an important source of salmonellosis [16,17]. In the Gallus sector in France, *Campylobacter* and *Salmonella* are both present at the slaughterhouse with an overall prevalence of 87.5% and 7.52% on carcasses, respectively [7,18], and at the retail level [19,20]. The current European regulations that were laid down to control these two pathogens at the slaughterhouse level have imposed a total absence of *Salmonella* spp. since 2010 [21] and, since 2018, a process hygiene criterion (m < 1000 CFU/g) for *Campylobacter* on broiler carcasses (neck skin) [22]. Both bacteria can be present on broiler meat; however, in the largest study performed in France on 58 slaughterhouses during a 12-month period, there was no correlation between *Salmonella* and *Campylobacter* prevalence on chicken carcasses [20]. A recent opinion issued by the European Food Safety Authority recommends research and field studies involving these two bacteria concomitantly for a better understanding of their behavior and for a better control in the field [23]. *Salmonella* is a facultative anaerobe bacterium, whereas *Campylobacter* has relatively stringent growth requirements. *Campylobacter* spp. are thermotolerant and typically grow at 37 °C, but the high optimum growth temperature of *C. jejuni* is 42 °C. Most *Campylobacter* species are microaerophilic, optimally growing in low oxygen such as an atmosphere of 5% oxygen, 10% carbon dioxide and 85% nitrogen [13,24]. *Campylobacter* has already shown prolonged survival when co-cultured with *Pseudomonas* spp., allowing it to cope with adverse environmental conditions under normal aerobic conditions [25]. Similar results were also observed by a few studies that investigated the co-culture of *Campylobacter* with other protozoa and bacteria under the same conditions, such as *Acanthamoeba castellanii*, *Tetrahymena pyriformis*, *Staphylococcus aureus* and *Escherichia coli* [26,27,28,29,30]. Moreover, *Campylobacter* spp. have already shown a significant correlation with *Blastocystis* sp., suggesting that the presence of *Campylobacter* spp. may be promoted by the presence of *Blastocystis* sp. and, similarly, that the absence of one is associated with the absence of the other [31]. The bacterium–bacterium interaction might set the basis for the survival of *Campylobacter jejuni* in foods and in human infection. Thus, it would be interesting to evaluate the co-culture of *C. jejuni* with other microorganisms.

This study aimed to investigate the survival ability of *Campylobacter jejuni* when co-cultured with *Salmonella* spp. under controlled aerobic conditions in order to assess the potential interactions that might occur when these two bacteria are simultaneously present.

## 2. Results

### 2.1. Survival of C. jejuni Co-Cultured with Salmonella spp. as a Function of Different C. jejuni Inoculum Levels

**Figure 1:**
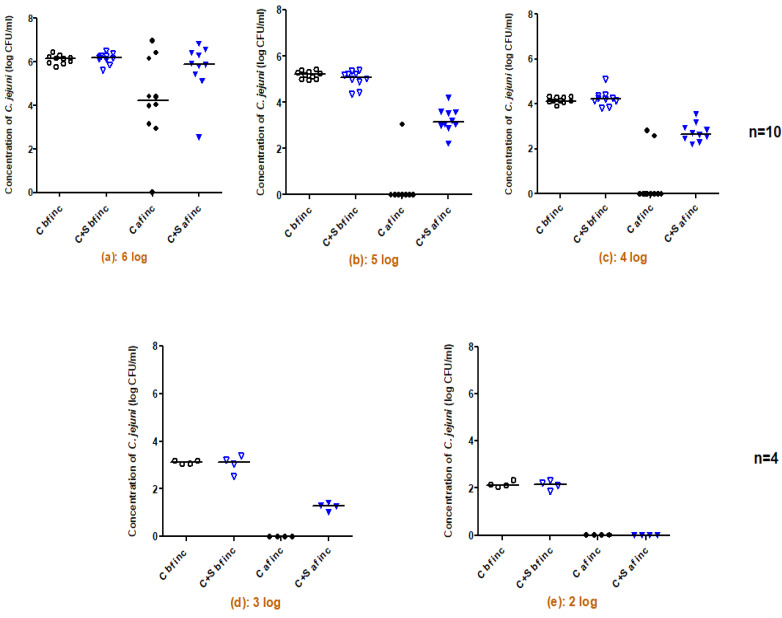
*C. jejuni* C97Anses640 counts before and after incubation under aerobic conditions (with or without *Salmonella* Blegdam) (n = 10: (**a**–**c**) from 6 to 4 log CFU/mL, n = 4: (**d**,**e**) for 3 and 2 log CFU/mL). C bf inc: Initial concentration of C97Anses640 before incubation; C + S bf inc: Initial concentration of C97Anses640 co-cultured with *S*. Blegdam before incubation; C af inc: Final concentration of C97Anses640 after aerobic incubation; C + S af inc: Final concentration of C97Anses640 co-cultured with *S*. Blegdam after aerobic incubation.

Effect of the initial concentration of *C. jejuni* as well as the impact of the presence of *Salmonella* on *C. jejuni* survival under aerobic conditions was studied based on an analysis of the enumeration results of each of the five concentrations of *C. jejuni* C97Anses640 (2, 3, 4, 5, and 6 log CFU/mL) grown alone or in co-culture with 10 CFU of *S*. Blegdam, before and after incubation for 16 h under aerobic conditions at 37 °C. The results shown in Figure 1 indicate that incubation of *C. jejuni* C97Anses640 alone under aerobic conditions for 16 h affected the level of surviving bacteria in an initial concentration-dependent manner. For instance, with the highest initial concentration of 6 log CFU/mL, the surviving bacteria varied from 0 to 6.9 log CFU/mL with a mean of 4.2 ± 2 log CFU/mL after aerobic incubation, but the difference between trials was not significant (*p* = 0.06) (Figure 1). However, significant reductions in *C. jejuni* concentrations were observed at lower initial concentrations: 0.4 ± 1 log CFU/mL (*p* = 0.0003) surviving bacteria were enumerated for an initial concentration of 5 log CFU/mL and 0.5 ± 1 log CFU/mL (*p* = 0.0001) for an initial concentration of 4 log CFU/mL (Figure 1). No survival was observed for the initial concentrations of 3 and 2 log CFU/mL (*p* = 0.02). These results observed with *C. jejuni* grown under aerobic conditions were not surprising due to its microaerophilic ability. On the other hand, the results clearly show that the survival of *C. jejuni* was positively affected by the presence of *S.* Blegdam during aerobic incubation. The improved survival was observed for the initial concentrations of 5 log CFU/mL, with 3.2 ± 0.5 log CFU/mL (*p* = 0.001) surviving *C. jejuni* bacteria enumerated when co-cultured with *S*. Blegdam, whereas only 0.4 ± 1 log CFU/mL of *C. jejuni* were recovered when incubated alone. Similarly, for an initial concentration of 4 log CFU/mL, 2.7 ± 0.4 log CFU/mL (*p* = 0.002) surviving *C. jejuni* bacteria were enumerated when co-cultured with *S*. Blegdam, whereas 0.5 ± 1 log CFU/mL of *C. jejuni* were recovered when incubated alone. Furthermore, 1.3 ± 0.2 log CFU/mL (*p* = 0.02) of surviving *C. jejuni* were enumerated in the co-culture for an initial concentration of 3 log CFU/mL of *C. jejuni*, but *C. jejuni* survived when cultured alone with this inoculum under the same aerobic conditions.

**Figure 2:**
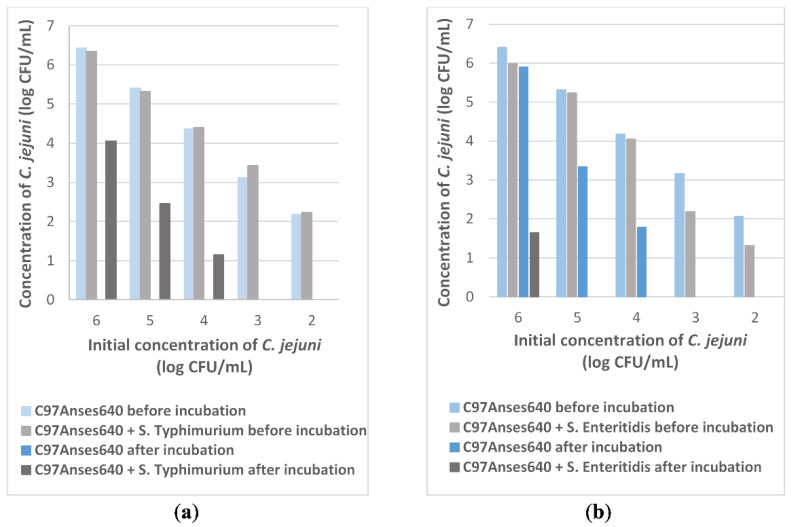
*C. jejuni* C97Anses640 counts before and after incubation under aerobic conditions. (**a**) With or without *Salmonella* Typhimurium; (**b**) With or without *Salmonella* Enteritidis (n = 1).

To further investigate the results obtained with *S.* Blegdam on the survival of *C. jejuni*, *S*. Typhimurium and *S*. Enteritidis, the two major serovars in poultry, were tested for their effect on *C. jejuni* survival following the same schemes as for *S*. Blegdam. The results are shown in Figure 2. In the co-culture of *C. jejuni* with *S.* Typhimurium, shown in Figure 2a, *C. jejuni* survival was positively affected by the presence of *S.* Typhimurium under aerobic conditions. In fact, the three samples with initial concentrations of 6, 5 and 4 log CFU/mL, 4 log CFU/mL, 2.5 log CFU/mL and 1.1 log CFU/mL of *C. jejuni* were enumerated, respectively, after incubation in the co-culture of *C. jejuni* with *S.* Typhimurium. No surviving bacteria were observed when cultured alone under the same aerobic conditions. No survival was observed for the low initial concentrations of 3 and 2 log CFU/mL, as previously observed with *S*. Blegdam. An enhancement in survival was not observed in the co-culture of *C. jejuni* with *S*. Enteritidis, as shown in Figure 2b, where 1.6 log CFU/mL of *C. jejuni* were observed only for the high initial concentration of 6 log CFU/mL, whereas 5.8 log CFU/mL were enumerated when cultured alone from the same initial concentration.

These trials demonstrated that when *C. jejuni* C97Anses640 was co-cultured with *S*. Blegdam at 37 °C under aerobic conditions, a beneficial effect on *C. jejuni* survival was observed, and its survival depended on the initial *C. jejuni* concentration. Moreover, this survival effect appeared to vary with different *Salmonella* serovars.

### 2.2. Survival of C. jejuni Co-Cultured with Salmonella spp. as a Function of Different Strains

To confirm the effect of *Salmonella* serovars on *C. jejuni* survival in co-culture, five different *C. jejuni* strains of the main prevalent *C. jejuni* genotypes (ST-21, ST-45, ST-464, ST-206 and ST-257) and the five *Salmonella* serovars regulated in poultry production (*S*. Typhimurium, *S*. Enteritidis, *S*. Infantis, *S*. Hadar and *S*. Virchow) were co-cultured under the same aerobic conditions. In this part of the investigation, one initial concentration of the *C. jejuni* strains (4 log CFU/mL) was used.

**Figure 3:**
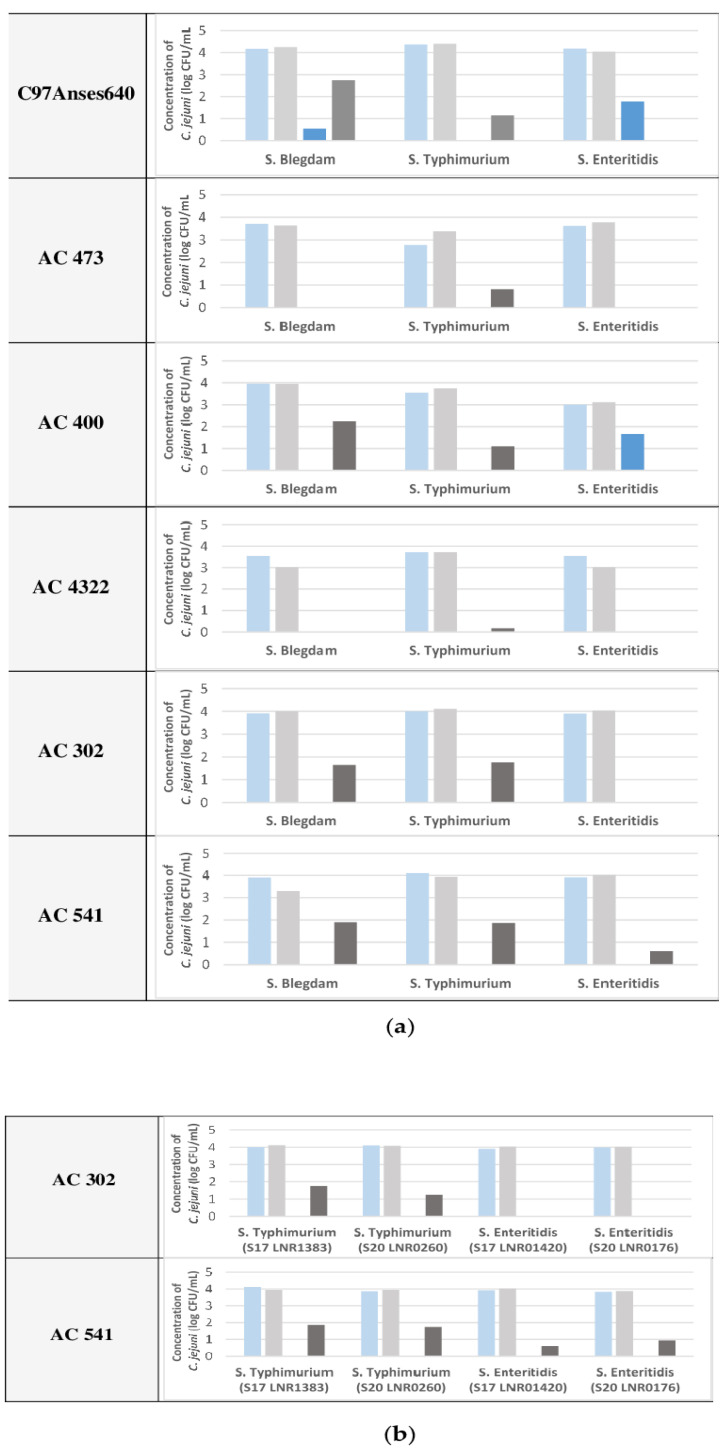
*C. jejuni* strain counts before and after incubation of 4 log CFU/mL under aerobic conditions with or without different *Salmonella* serovars (light blue bars: initial concentration of *C. jejuni*; light gray bars: initial concentration of *C. jejuni* co-cultured with *Salmonella*; dark blue bars: final concentration of *C. jejuni* following aerobic incubation; dark gray bars: final concentration of *C. jejuni* co-cultured with *Salmonella* following aerobic incubation). (**a**) Six strains of *C. jejuni* were co-cultured with or without *S*. Blegdam 421, *S*. Typhimurium (S17 LNR1383) and *S*. Enteritidis (S17 LNR01420); (**b**) *C. jejuni* strains AC 302 and AC 541 co-cultured with or without *S*. Typhimurium (S20 LNR0260) and *S*. Enteritidis (S20 LNR0176) compared to *S*. Typhimurium (S17 LNR1383) and *S*. Enteritidis (S17 LNR01420).

The results of all these co-cultures after incubation under aerobic conditions are presented in Figure 3a.

The results showed *C. jejuni* survival upon co-culture with *S*. Blegdam, *S*. Typhimurium and *S*. Enteritidis with differences in its survival depending on the *C. jejuni* strain used. All five *C. jejuni* strains tested survived in the co-culture with *S*. Typhimurium, where 1.9, 1.7, 1.1, 0.8 and 0.2 log CFU/mL were observed for the AC 541, AC 302, AC 400, AC 473 and AC 4322 strains, respectively. Three tested *C. jejuni* strains showed survival in the co-culture with *S*. Blegdam, where 2.2, 1.9 and 1.7 log CFU/mL of *C. jejuni* were recorded for the AC 400, AC 541 and AC 302 strains, respectively. In co-culture with *S*. Enteritidis, only the *C. jejuni* strain AC 541 showed a survival of 0.5 log CFU/mL. However, no survival was observed for any of the different combinations of co-cultures of *C. jejuni* with *S*. Infantis or *S*. Hadar (data not shown). In the co-culture with *S*. Virchow, for the first series where all five of the *C. jejuni* strains were tested on mCCD plates, *C. jejuni* survival was not possible to estimate because *S*. Virchow grew on the *Campylobacter* mCCD plates. However, when this co-culture was tested on CASA agar with the *C. jejuni* strain that showed significant survival in the co-culture with *S*. Blegdam, *S*. Typhimurium and *S*. Enteritidis, no survival was observed for *C. jejuni* with *S*. Virchow. To pursue the investigation of the impact of *Salmonella* on *C. jejuni* survival, one other strain from a serovar of *S*. Typhimurium (S20LNR0260) and *S*. Enteritidis (S20LNR0176) was tested with two *C. jejuni* strains that showed either significant survival (AC 541) or no survival (AC 302). One concentration of *C. jejuni* (4 log CFU/mL) was used and the results were compared with the results obtained above and are presented in Figure 2b. These results showed that with the two tested *S*. Enteritidis strains, the same *C. jejuni* strain (AC 541) survived, and with the two *S*. Typhimurium strains, both *C. jejuni* strains (AC 302 and AC 541) were noted.

These trials demonstrated that *C. jejuni* survival depends on the specific *Salmonella* serovar and on the specific *C. jejuni* strain when co-cultured under aerobic conditions; *C. jejuni* showed enhanced survival in the presence of *S*. Blegdam, *S*. Typhimurium and, to a lesser extent, *S*. Enteritidis, but no survival was observed in the presence of *S*. Hadar, *S*. Infantis or *S*. Virchow. Moreover, *C. jejuni* survival was reproducible when testing different strains from *Salmonella* serovars that showed a positive effect on *C. jejuni* survival.

### 2.3. Effect of C. jejuni on Salmonella Growth under Aerobic Conditions

In all the tested combinations of co-cultures between *Salmonella* and *C. jejuni* strains, the effect of *C. jejuni* on *Salmonella* growth under aerobic conditions was also assessed. Analyzing the same samples from all of these trials, the combinations showed that the different initial conditions of *C. jejuni* (2, 3, 4, 5, and 6 log CFU/mL) had no effect on *Salmonella* growth (data not shown) and that the presence of different *C. jejuni* strains had similarly no impact on the growth of the different *Salmonella* serovars and strains; the results are presented in Table 1. All samples showed comparable results, with mean enumeration concentrations of 8.5 ± 0.09 log CFU/mL after incubation during 16 h in aerobic conditions at 37 °C. This observation indicates that all of the *Salmonella* serovars and strains tested in this study grew in the same way in the absence or presence of different concentrations of *C. jejuni* regardless of the tested *C. jejuni* strain in the culture medium.

## 3. Discussion

The polymicrobial colonization of retail chicken meat or products is common [32,33], and can have antagonistic or synergistic effects on foodborne pathogens [34]. To date, information on the interaction between bacteria is of increasing importance for public health and food safety measures. *Campylobacter* and *Salmonella* are the two most commonly reported causes of bacterial gastroenteritis in many high-income countries, affecting millions of people every year, and poultry is generally recognized as the main source of infection. Consequently, the contamination of chicken meat or products with these two bacteria occurs despite the food safety measures and strategies to combat these diseases, which have shown limited success.

In this paper the authors focused on the interaction between *C. jejuni* and *Salmonella* spp. under atmospheric conditions; we highlighted an aspect of *C. jejuni* whereby it benefits from the presence of *Salmonella* spp. to survive. Thus, the interaction between these two bacteria was examined, showing how *C. jejuni* can survive under stressful aerobic conditions in the poultry-processing chain, despite the fact that it is an obligate microaerophile, meaning that it usually cannot survive in aerobic conditions.

Despite the general perception that *C. jejuni* is microaerophilic and therefore sensitive to oxygen, our data showed that *C. jejuni* C97Anses640 was able to survive alone under aerobic conditions, particularly at the highest initial concentration of 6 log CFU/mL, but not at the lower concentrations. Some previous studies have already reported the ability of *C. jejuni* to survive in the presence of oxygen at a similar high initial concentration, attributing survival to oxygen playing a growth-limiting role in high-concentration bacterial cultures, but being toxic in low-concentration cultures under aerobic conditions. Therefore, these two factors may be involved in the absence of growth observed in low-concentration cultures [35,36,37]. In addition, these studies correlated survival to alterations in gene expression in response to *C. jejuni* concentrations and to interbacterial interactions that thus contribute to the enhanced ability of bacteria to survive environmental changes. Other studies have indicated that *C. jejuni* contains a range of enzymes involved in oxidative stress resistance. For example, one study characterized a unique human isolate of *C. jejuni*, named Bf, that can grow under aerobic conditions suggesting that this isolate is highly resistant to oxidative stress conditions [38].

In addition, our results clearly showed that the survival of *C. jejuni* C97Anses640 was positively affected by the presence of *S*. Blegdam during aerobic incubation. This enhanced survival was also observed for high and low initial concentrations (i.e., 5, 4 and 3 log CFU/mL). These results therefore suggest that *S*. Blegdam has a beneficial effect on *C. jejuni* C97Anses640 survival under aerobic conditions, even in low initial concentrations of *C. jejuni* C97Anses640. In addition, similar *C. jejuni* C97Anses640 survival was observed in co-culture with *S*. Typhimurium in the same conditions, which highlighted and confirmed *C. jejuni* C97Anses640 survival in conjunction with *Salmonella* spp. This protective effect of co-culture has been previously suggested in several studies on amoebas, demonstrating the ability of *C. jejuni* to interact with and be protected by *A. polyphaga* [29,39,40,41]. Moreover, survival of *Campylobacter* spp. has been observed in conjunction with different species of the genus *Acanthamoaeba* [27,28]. Similarly, the presence of *Tetrahymena pyriformis* and *A. castellanii* in co-culture with *C. jejuni* can significantly delay the decline of *C. jejuni* viability and significantly increase *C. jejuni* resistance under aerobic conditions [42]. Furthermore, other reports have suggested that *C. jejuni* survival in the presence of *A. castellanii* is due to the depletion of dissolved oxygen by *A. castellanii*, creating the microaerobic conditions that may be beneficial to *C. jejuni*. Another study showed that the co-culture of *A. castellanii* and *C. jejuni* delays the decline in and increases the long-term survival of *C. jejuni* [26,43]. These results also corroborate a study that showed that *C. jejuni* may benefit from the low-oxygen environment of epithelial cells to survive there [44]. In contrast, our results contradict a previous study that showed that *C. jejuni* can survive independently of the presence of *A. castellanii* [45]. Another possible mechanism of dealing with high oxygen concentrations may be metabolic commensalism with aerobic microorganisms found in foods. For instance, *C. jejuni* can survive by metabolic commensalism under conditions of atmospheric oxygen concentrations with the support of *Pseudomonas* spp. [25]. Furthermore, a recent study reported the survival of *C. jejuni* strains in the presence of *S. aureus* in low-temperature and aerobic conditions, indicating that metabolites produced by *S. aureus* and the reduced levels of dissolved oxygen improve the survival of *C. jejuni* [30]. Additionally, a similar relationship may exist between *C. jejuni* and *Salmonella* spp.

On the other hand, our results indicated that *C. jejuni* survival depended both on the *Salmonella* serovar and the specific *C. jejuni* strain when co-cultured under aerobic conditions. Neither *S*. Hadar nor *S*. Infantis improved *C. jejuni* survival regardless of the *C. jejuni* strain, whereas all tested *C. jejuni* strains were positively affected by the presence of *S*. Typhimurium. However, the results varied according to the *C. jejuni* strain when co-cultured with *S*. Blegdam and *S*. Enteritidis. This finding corroborates a previous study in which *C. jejuni* strains isolated from chicken meat co-cultured with different *Pseudomonas* species displayed increased aerobic tolerance, but differed in their overall extent of tolerance depending on the tested strain [25]. Similarly, another study suggested that not all *Acanthamoeba* species have the same effect and that the specific *C. jejuni* strain has an impact on *C. jejuni*–*Acanthamaeba* interactions [43]. Although most *C. jejuni* strains have been shown to be sensitive to the toxic effects of oxygen concentrations of up to 21%, each individual strain has a different oxygen tolerance. This latter study clearly demonstrated differences in responses among *C. jejuni* strains to environmental stresses [46].

In the present study, *Salmonella* serovars and strains were not affected by *C. jejuni* after co-culture under aerobic conditions, regardless of the initial *C. jejuni* concentration and strain tested in the culture medium. Earlier studies showed similar results in which the presence of *C. jejuni* in co-culture with *A. castellanii* did not affect *A. castellanii* growth after incubation in the same conditions [43]. The survival of *C. jejuni* after co-culture with *Salmonella* is consistent with our hypothesis that *Salmonella* can mediate the survival of *C. jejuni* during transmission in the food chain or the environment. Moreover, we suspect that the presence of *Salmonella* provides a microaerobic environment suitable for *C. jejuni* survival or even growth, but additional work is needed to decipher this interaction mechanism.

This original work emphasizes the need to obtain more data about the co-infection of poultry by both pathogens in conventional flocks as recommended by a recent EFSA opinion [23]. Experimental studies would be required to assess the interaction between *Campylobacter* and *Salmonella* in field trials and consequently to adapt the control measures.

## 4. Materials and Methods

### 4.1. Bacterial Strains and Culture Conditions

All the strains used in this study belong to the collection of ANSES laboratory. For *C. jejuni* strains, *C. jejuni* C97Anses640 and different strains covering the main genotypes found in poultry (strains AC 473 (ST-21), AC 400 (ST-45), AC 4322 (ST-464), AC 302 (ST-206) and AC 541 (ST-257)) [47] were used. For *Salmonella***,** *Salmonella* Blegdam 421 and strains representing the major *Salmonella* serovars targeted in poultry production regulations (*S*. Typhimurium (S17 LNR1383 and S20 LNR0260), *S*. Enteritidis (S17 LNR01420 and S20 LNR0176), *S*. Infantis (S20 LNR0009), *S*. Hadar (S20 LNR0028) and *S*. Virchow (S19 LNR0182)) were used.

*C. jejuni* strains were subcultured from stock solutions stored at –80 °C by cultivating them on blood agar (GS, Thermo Fisher Diagnostics, Illkirch-Graffenstaden, France) and modified charcoal–cefoperazone–deoxycholate (mCCD, Thermo Fisher Diagnostics, Dardilly, France) agar. All plates were incubated at 41.5 °C for 48 h under microaerobic conditions (5% O_2_, 10% CO_2_ and 85% N_2_) in a jar equipped with a microaerobic atmosphere generating kit (Labo and CO, Whitley jar gassing system, Marolles-En-Brie, France) or with a CampyGen sachet (Thermo Scientific, Tokyo, Japan). One single characteristic colony of *C. jejuni* was picked from mCCD agar to inoculate 5 mL of Brucella Broth (Becton, Dickinson and Company, Le Pont-de-Claix, France). After microaerobic incubation at 41.5 °C for 24 h, 100 µL were added into another 5 mL of Brucella Broth and incubated microaerobically at 41.5 °C for 18 h. Serial dilutions (1:10) were then carried out to generate bags for the survival experiments (Section 4.2) with:

- different final concentrations of *C. jejuni* ranging from 2 to 6 log CFU/mL to study the survival of *C. jejuni* co-cultured with *Salmonella* spp. as a function of different *C. jejuni* inoculum levels.
- a final concentration of 4 log CFU/mL to study the survival of *C. jejuni* co-cultured with *Salmonella* spp. as a function of different strains.

After these dilutions, 100 µL of diluted *C. jejuni* Brucella Broth were plated in duplicate on mCCD agar and the plates were incubated microaerobically at 41.5 °C for 48 h.

All the selected *Salmonella* strains were directly used from frozen aliquots containing 10 CFU/mL preserved in glycerol peptone broth (Fisher Scientific, Thermo Fisher Diagnostics, Dardilly, France) and stored at –80 °C. These aliquots were defrosted then added to buffered peptone water (BPW) bags (bioMérieux, Marcy-l’Etoile, France) during the survival experiments (Section 4.2).

### 4.2. Survival Assays

**Figure 4:**
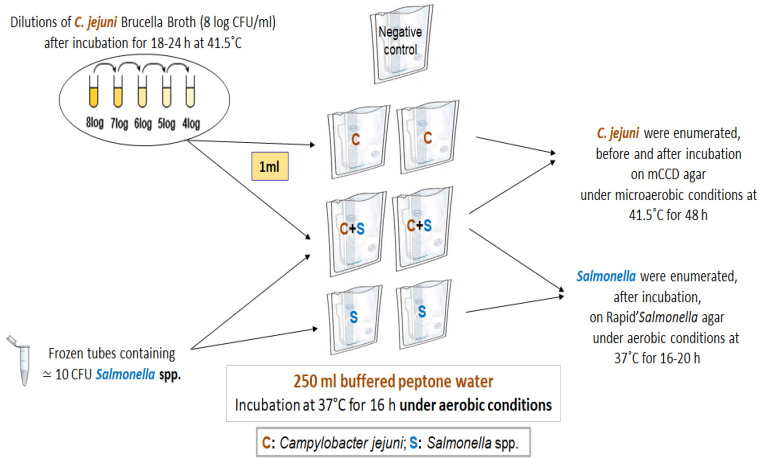
Experimental protocol for survival assays: A culture of *C. jejuni* (from 8 to 4 log CFU/mL) was diluted to inoculate the bags, containing 250 mL buffered peptone water, with different final concentrations of *C. jejuni* ranging from 6 to 2 log CFU/mL. Then, *C. jejuni* obtained in the bags were cultured under aerobic conditions at 37 °C for 16 h in the presence or absence of 10 CFU of *Salmonella* spp.

The survival assays were performed following the procedure described in Figure 4. Briefly, bags containing 250 mL BPW were inoculated with (i) a *C. jejuni* strain (at different final concentrations, ranging from 2 to 6 log CFU/mL), (ii) a *Salmonella* serovar (at 10 CFU of different *Salmonella* serovars) or (iii) *C. jejuni* and *Salmonella* simultaneously (at different concentrations of *C. jejuni* and 10 CFU of different *Salmonella* serovars). All experiments included a negative control bag containing 250 mL BPW. All bags were then cultured under aerobic conditions at 37 °C for a total of 16 h.

The differences regarding the survival of *C. jejuni* when cultured individually or in co-culture with *Salmonella* were assessed by enumerating the solutions from different bags. For all experiments, 10 mL were removed from each bag prior to serial dilutions in tryptone salt broth (bioMérieux, Bruz, France). One ml of the first dilution was distributed on three plates of mCCD agar and then 100 µL of each of the following dilutions were plated in duplicate on mCCD agar. Colonies were counted after microaerobic incubation at 41.5 °C for 48 h. This same experiment was repeated on *Campylobacter* Selective Agar (CASA, bioMérieux, Craponne, France) for *S*. Virchow due to the growth of *S*. Virchow on *Campylobacter* mCCD agar.

The impact of *C. jejuni* on *Salmonella* growth was also assessed after the enumeration of *Salmonella* grown individually and in co-culture with *C. jejuni* after the incubation of the bags. Ten mL were removed from each bag prior to serial dilutions in tryptone salt broth and then 100 µL of the following dilutions were plated in duplicate on Rapid’*Salmonella* plates (Rapid’*Salmonella*, Bio-Rad, Marnes-La-Coquette, France). Colonies were counted after aerobic incubation at 37 °C for 24 h.

The results were expressed in terms of either the surviving bacteria or the reduction in bacterial concentration. The concentration of surviving bacteria is the count of *C. jejuni* recovered and enumerated on mCCD plates, after incubation at 37 °C for 16 h under aerobic conditions.

The reduction in bacterial concentration is the difference between the initial concentration of the *C. jejuni* inoculum before incubation and the concentration of the *C. jejuni* enumerated on mCCD plates after incubation at 37 °C for 16 h under aerobic conditions.

### 4.3. Statistical Analyses

In all the experiments, for *C. jejuni* counts, only the plates with less than 150 colonies were counted, the mean CFU/mL was calculated, and viable counts were log10-transformed for further analysis. The experiments on the effect of *C. jejuni* C97Anses640 inoculum and *S*. Blegdam on *C. jejuni* survival under aerobic conditions were repeated 10 times for the high initial concentrations of 6, 5 and 4 log CFU/mL and four times for the two low initial concentrations of 3 and 2 log CFU/mL. A nonparametric Mann–Whitney test (performed using GraphPad Prism version 9) was used to compare the experimental groups, the *C. jejuni* C97Anses640 count before aerobic incubation with the *C. jejuni* C97Anses640 count after aerobic incubation in both cases, when incubated alone and in the presence of *S*. Blegdam. Similarly, a nonparametric Mann–Whitney test (using GraphPad Prism version 9), was used to compare differences regarding the survival of *C. jejuni* when cultured individually or in co-culture with *S*. Blegdam under aerobic conditions. A *p* < 0.05 was used to indicate whether the differences were statistically significant.

## 5. Conclusions

This is the first study of *C. jejuni* (a microaerophilic bacterial species) co-cultured with *Salmonella* spp. (facultative anaerobic bacterial species), demonstrating that *C. jejuni* shows significant survival in the co-culture with *Salmonella* spp. under aerobic conditions. These results suggest potential interactions between *Salmonella* spp. and *C. jejuni* that require further investigation for a clearer understanding of the behavior of these pathogens in natural habitats, such as in live animals and in poultry meat products. Given that most strategies to reduce campylobacteriosis have not been successful, new approaches in regard to microorganism-microorganism interactions—and thus different control strategies—are needed to fight this major zoonotic pathogen.

## Figures and Tables

**Table 1:** Counts of *Salmonella* serovars and strains following incubation in the presence or absence of different *C*. *jejuni* strains under aerobic conditions. *Salmonella* (10 CFU) were incubated in the presence or not of 4 log CFU/mL of various *C. jejuni* strains for 16 h.

		*Salmonella* Counts (log CFU/mL)						
		No *C. jejuni*	*C. jejuni* Strains					
*Salmonella* Serovars and Strains			AC302	AC 400	AC 473	AC 541	AC 4322	C97Anses640
*S.* Blegdam		8.6	8.5	8.5	8.5	8.5	8.5	8.8
*S.* Typhimurium	S17LNR1383	8.5	8.5	8.5	8.5	8.6	8.5	8.5
	S20LNR0260	8.5	8.5	-	-	8.5	-	-
*S.* Enteritidis	S17LNR01420	8.5	8.5	8.5	8.5	8.5	8.5	8.6
	S20LNR0176	8.4	8.5	-	-	8.4	-	-
*S*. Infantis		8.5	8.5	8.5	8.6	8.6	8.5	8.8
*S*. Hadar		8.7	8.6	8.6	8.7	8.7	8.7	8.8
*S*. Virchow		8.6	8.2	8.0	8.7	8.5	8.7	8.6

## Data Availability

Not applicable.
